# Elevated RANTES levels are associated with increased risk of cerebral atherosclerotic stenosis

**DOI:** 10.1186/s12883-023-03079-9

**Published:** 2023-01-25

**Authors:** Yinping Guo, Qianqian Kong, Yi Zhang, Jing Zhao, Zhiyuan Yu, Dan He, Hao Huang, Xiang Luo

**Affiliations:** 1 Department of Neurology, Tongji Hospital, Tongji Medical College, Huazhong University of Science and Technology, Wuhan, Hubei 430030 P.R. China; 2 Department of Neurology, Guangdong Provincial Key Laboratory for Diagnosis and Treatment of Major Neurological Diseases, National Key Clinical Department and Key Discipline of Neurology, The First Affiliated Hospital, Sun Yat-Sen University, Guangzhou, Guangdong China

**Keywords:** Cerebral atherosclerotic stenosis (CAS), Cytokines, RANTES

## Abstract

**Background:**

Cerebral atherosclerotic stenosis (CAS) is a significant factor in the development of acute ischemic stroke (AIS). Previous studies have reported that cytokines are involved in atherosclerotic diseases, although the relationship between serum levels of the chemokine RANTES (regulated on activation, normal T-cell expressed and secreted) and the presence of CAS remains unclear.

**Methods:**

In total, 127 participants (65 non-AIS controls and 62 patients with AIS) were involved in this study. CAS was defined as the presence of ≥ 50% stenosis in major intracranial or extracranial artery by a Digital Substraction Angiography (DSA) examination, and we classified all participants into four groups according to stroke and CAS status. Serum concentrations of 8 cytokines, including RANTES, were measured by the Human ProcartaPlex Multiplex Immunoassay Kit.

**Results:**

Seventy-eight participants (61.41%) had CAS, of which 39 cases with AIS and 39 case with non-AIS. Patients with CAS had higher RANTES levels compared to non-CAS patients in both the non-AIS group (10.54 ± 0.80 vs. 13.20 ± 0.71, *p* = 0.016) and stroke group (11.96 ± 0.87 vs. 15.03 ± 0.75, *p* = 0.011), and multivariate logistic regression analysis showed that the RANTES level is independently associated with CAS in both the non-AIS group (adjusted odds ratio (OR), 1.07; 95% CI, 1.02–1.12, *P* = 0.004) and stroke group (adjusted OR, 1.32; 95% CI, 1.10–1.58, *P* = 0.003).

**Conclusion:**

Patients with CAS have higher levels of serum RANTES than non-CAS patients regardless of stroke status suggesting that RANTES may play an important role in the formation of CAS.

**Supplementary Information:**

The online version contains supplementary material available at 10.1186/s12883-023-03079-9.

## Introduction

Cerebrovascular disease is a major health problem worldwide and one of the three major causes of death [1, 2]. In cerebrovascular diseases, the incidence of ischemic stroke is as high as 87% and its disability rate exceeds 50% [3, 4]. Cerebral atherosclerotic stenosis (CAS) affects both intra- and extra-cranial arteries and is widely recognized as a critical risk factor for ischemic stroke [5]. Patients with CAS-related acute ischemic stroke often have poor outcomes and increased risk of stroke recurrence [6]. Prior studies have reported that 23% of ischemic strokes are caused by large artery atherosclerosis [7], and the risk of recurrent stroke in these patients is 11–33% and increases with the degree of vascular stenosis.[8–10] The Warfarin-Aspirin Symptomatic Intracranial Disease (WASID) trial reported a stroke recurrence rate of up to 17% despite medical intervention, leading to increased disability rates [11]. Identifying patients with a high risk of CAS is essential to the prevention of both occurrence and recurrence of stroke.

The chemokine regulated on activation, normal T-cell expressed and secreted (RANTES) is involved in the migration of inflammatory cells to the inner membrane and plays an important role in the formation of plaque [12]. In particular, RANTES recruits leukocytes to sites of inflammation, promotes lymphocyte and macrophage mobilization and chemotaxis, and further stimulates the release of mediators. Studies have shown significant correlations between RANTES levels and atherosclerotic plaque progression [12], markers of heart failure [13], and even acute coronary syndromes [14]. In a prospective study, elevated RANTES concentrations were used to predict the occurrence of ischemic stroke [15]. However, studies on the association between serum cytokine levels of RANTES and the risk of CAS are limited.

In this study, we aimed to assess the relationship between the concentration of serum RANTES and the presence of CAS, which may serve as a biomarker for identifying patients with high risk of CAS.

## Method

### Study population

We prospectively recruited acute ischemic stroke (AIS) and non-AIS control patients who were hospitalized in the Hospital from July 2019 to December 2019. AIS was diagnosed according to clinical symptoms and imaging (magnetic resonance/computer tomography) within 14 days of symptom onset. Non-AIS patients were hospitalized due to dizziness and headache, and did not suffer an acute stroke in the past 6 months. Exclusion criteria was (1) age ≤ 30 years; (2) incomplete neuroimaging data and laboratory tests; (3) prior thrombolysis or thrombectomy; (4) atrial fibrillation, cardioembolism and serious peripheral arterial disease; (5) recent local and systemic infectious disease(s); (6) intracranial and extracranial arterial stenosis caused by arterial dissection, arteritis, moyamoya disease, and muscle fiber dysplasia; and (7) a history of malignant tumors, severe liver or kidney dysfunction, or systemic disease. This study has been approved by the Tongji Hospital Ethics Committee (No. TJ-IRB20210107) and conducted according to the Declaration of Helsinki. All participants provided informed consent.

### Definition of CAS

Cerebrovascular images were obtained from all participants, and CAS was defined and determined by 50–99% stenosis or occlusion by digital subtraction angiography (DSA) examinations in at least one major arterial segment, including the intracranial or extracranial segment of the carotid artery (ICA) or vertebral artery (VA), M1 middle cerebral artery (MCA) and basilar artery (BA). A significantly stenotic vessel segment is considered a responsible CAS when an infarction lesion occurs in its blood supply area. In the current study, we assessed the location and severity of responsible CAS.

### Clinical assessments

Clinical data were collected from the clinical record and included age, gender, history of smoking and drinking, and medical history (e.g., prior ischemic stroke/TIA, coronary heart disease, hypertension, diabetes mellitus, hyperlipidemia). Hypertension was defined as systolic blood pressure (SBP) ≥ 140 mmHg and/or diastolic blood pressure (DBP) ≥ 90 mmHg and/or prior treatment with antihypertensive medication [16]. Diabetes mellitus was defined as glycosylated hemoglobin A1c (HbA1c) ≥ 6.5%, two-hour plasma glucose ≥ 11.1 mmol/L after an oral glucose tolerance test, or self-reported history of diabetes mellitus [11, 17, 18]. Laboratory data included serum levels of fasting triglycerides, total cholesterol, high-density lipoprotein cholesterol (HDL-C), low-density lipoprotein cholesterol (LDL-C), creatinine, glomerular filtration rate (eGFR), fasting plasma glucose (FPG), and HbA1c platelet indexes. All data were collected in a standard manner in the laboratory of the hospital within 24 h of enrollment.

### Measurement of serum cytokine level

Peripheral blood samples were collected within 24 h of admission with vacutainer tubes containing coagulant accelerator (Becton–Dickinson, San Jose, USA) and immediately centrifuged at 1,278 g for 15 min at 4 °C. Serum samples were stored at − 80 °C until analysis. The concentrations of serum cytokines (including TNF-α, IL-6, MMP-9, MMP-1, IP-10, RANTES VEGF, and IL-23,) were analyzed using the Human ProcartaPlex Multiplex Immunoassay Kit (Affymetrix, eBioscience, USA) according to the manufacturer’s instructions.

### Statistical analysis

Statistical analyses were performed using IBM SPSS 22.0 software (IBM Corp., Armonk, NY, USA), and *P* < 0.05 was considered statistically significant. Categorical variables are represented as frequencies (percentages), whereas continuous variables are represented as mean ± standard error of the mean (SEM) or median [interquartile range]. Non-AIS vs. AIS groups or non-CAS vs. CAS groups were compared using a chi-square test, two-sided *t*-test or Mann–Whitney *U*-test according to their distributions. The Kruskal–Wallis H test was utilized to explore the association between serum RANTES levels and CAS features. Multivariate logistic regression analyses were used to determine the relationship between serum RANTES levels and CAS. Odds ratio (OR) was adjusted by variables that had a significant clinical correlation (including age, gender, smoking history, drinking history, and medical history) and variables with *P* < 0.05 by univariate logistic regression analysis. Finally, receiver operating characteristic (ROC) curve analysis was used to determine whether serum RANTES predicts CAS, and the area under the ROC curve (AUC) is reported.

## Results

### Baseline characteristics and serum cytokines levels of participants

One hundred twenty-seven participants were included in the study analysis (65 [51.18%] AIS patients and 62 [48.82%] non-AIS controls). Thirty-nine (30.71%) AIS patients and 39 (30.71%) non-AIS controls with at least one segment of CAS, while 26 (20.47%) AIS patients and 23 (18.11%) non-AIS controls were absent of CAS. Baseline characteristics are illustrated in Table 1. Compared to controls, patients with acute ischemic stroke were younger overall, but demonstrated lower levels of both total cholesterol (TC) and high-density lipoprotein (HDL). No significant differences between baseline characteristics were found between the CAS and non-CAS groups in patients with or without AIS.

**Table 1:** Characteristics of participants according to stroke and CAS status

Variables	Non-AIS (*n* = 65)	AIS (*n* = 62)	*p*-value	Non-AIS			AIS		
				**Non-CAS (*n* = 26)**	**CAS (*n* = 39)**	***p*-value**	**Non-CAS (*n* = 23)**	**CAS (*n* = 39)**	***p*-value**
Age	59.95 ± 1.32	54.23 ± 1.48	**0.019**	59.85 ± 2.05	58.34 ± 1.75	0.581	53.13 ± 2.57	54.87 ± 1.82	0.575
Male, n (%)	45 (69.2)	50 (80.6)	0.139	15 (57.7)	30 (76.9)	0.100	18 (78.3)	32 (82.1)	0.715
Smoking, n (%)	28 (43.1)	26 (41.9)	0.879	8 (30.8)	20 (51.3)	0.102	7 (30.4)	19 (48.7)	0.159
Drinking, n (%)	25 (38.5)	23 (37.1)	0.874	9(34.6)	16 (41.0)	0.603	5 (34.8)	15 (38.5)	0.772
**Medical history, n (%)**									
Prior stroke/TIA	11 (16.9)	10 (16.1)	0.904	2 (7.7)	9 (23.1)	0.177	3 (13.0)	7 (17.9)	0.731
CAD	5 (7.7)	7 (11.3)	0.488	3 (11.5)	2(5.1)	0.382	4 (17.4)	3 (7.7)	0.408
Hypertension	40 (61.5)	37 (59.7)	0.830	16 (61.5)	24 (61.5)	1.000	13 (56.5)	24 (61.5)	0.791
Diabetes mellitus	19 (29.2)	13 (21.0)	0.284	5 (19.2)	14 (35.9)	0.148	5 (21.7)	8 (20.5)	0.909
Hyperlipidemia	6 (9.2)	7 (11.3)	0.702	2 (7.7)	4 (10.3)	1.000	4 (17.4)	3 (7.7)	0.408
**Laboratory findings**									
TC, mmol/L	3.67 ± 0.12	3.27 ± 0.11	**0.019**	3.80 ± 0.18	3.59 ± 0.17	0.422	3.36 ± 0.19	3.22 ± 0.14	0.552
Triglyceride, mmol/L	1.43 ± 0.13	1.39 ± 0.12	0.830	1.43 ± 0.15	1.43 ± 0.19	0.989	1.43 ± 0.21	1.37 ± 0.14	0.783
HDL-C, mmol/L	1.02 ± 0.03	0.91 ± 0.03	**0.005**	1.06 ± 0.04	0.99 ± 0.04	0.218	0.90 ± 0.04	0.91 ± 0.03	0.824
LDL-C, mmol/L	2.27 ± 0.11	1.99 ± 0.10	0.061	2.33 ± 0.16	2.22 ± 0.14	0.603	2.06 ± 0.80	1.95 ± 0.12	0.594
Creatinine, µmol/L	72.71 ± 2.25	73.22 ± 2.43	0.878	72.0 ± 3.21	73.19 ± 3.12	0.798	72.57 ± 3.51	73.61 ± 3.30	0.838
eGFR, ml/min/1.73m^2^	92.43 ± 1.93	95.76 ± 2.18	0.254	90.69 ± 2.51	95.60 ± 2.77	0.464	97.19 ± 3.27	94.91 ± 2.89	0.616
Fasting glucose, mmol/L	5.76 ± 0.24	5.84 ± 0.29	0.826	5.74 ± 0.39	5.77 ± 0.32	0.941	5.87 ± 0.59	5.83 ± 0.31	0.950
HbA1c, %	6.46 ± 0.20	6.44 ± 0.22	0.936	6.28 ± 0.27	6.58 ± 0.27	0.463	6.34 ± 0.34	6.50 ± 0.30	0.719
Platelet count (× 10^9^/L)	221.92 ± 8.01	221.73 ± 7.44	0.987	221.8 ± 13.5	222.7 ± 10.1	0.908	221.16 ± 9.96	221.16 ± 10.32	0.920

Data presented as mean ± SEM or *n* (%)

Abbreviations: *CAS* Cerebral artery stenosis, *AIS* Acute ischemic stroke, *TIA* Transient ischemic attack, *TC* Total cholesterol, *HDL-C* High-density lipoprotein cholesterol, *LDL-C* Low-density lipoprotein cholesterol, *eGFR*, Glomerular filtration rate, *HbAc1* Glycosylated hemoglobin A1c

AIS patients had higher levels of MMP-1 and RANTES compared to non-AIS patients (*p* < 0.05), and in patients with AIS, those with CAS had higher levels of RANTES and IP-10. Non-AIS controls with CAS, however, had higher levels of RANTES and IL-23 (Table 2).

**Table 2:** Characteristics of participants according to stroke and CAS status

Variables	Non-AIS (*n* = 65)	AIS (*n* = 62)	*p*-value	Non-AIS			AIS		
				**Non-CAS (*n* = 26)**	**CAS (*n* = 30)**	***p*-value**	**Non-CAS (*n* = 23)**	**CAS (*n* = 38)**	***p*-value**
TNF-α, pg/ml	5.42 ± 0.23	5.56 ± 0.21	0.654	5.70 ± 0.34	5.24 ± 0.30	0.325	5.50 ± 0.38	5.6 ± 0.26	0.823
IL-6, pg/ml	3.29 ± 0.18	3.6 ± 0.21	0.253	3.26 ± 0.23	3.30 ± 0.26	0.890	3.20 ± 0.36	3.85 ± 0.26	0.146
MMP-9, ng/ml	27.61 ± 4.89	35.09 ± 5.93	0.331	25.64 ± 4.83	28.88 ± 7.44	0.749	31.09 ± 9.38	37.45 ± 7.69	0.609
MMP-1, ng/ml	1.80 ± 0.15	3.03 ± 0.39	**0.003**	1.95 ± 0.25	1.71 ± 0.19	0.446	2.41 ± 0.36	3.40 ± 0.58	0.226
IP-10, pg/ml	31.98 ± 2.11	26.96 ± 2.19	0.101	26.29 ± 2.25	35.88 ± 3.08	**0.025**	28.16 ± 3.94	26.28 ± 2.63	0.684
RANTES, ng/ml	12.12 ± 0.55	13.89 ± 0.60	**0.031**	10.54 ± 0.80	13.20 ± 0.71	**0.016**	11.96 ± 0.87	15.03 ± 0.75	**0.011**
VEGF, pg/ml	51.57 ± 5.55	52.16 ± 4.57	0.935	52.28 ± 7.04	51.10 ± 8.05	0.918	52.25 ± 5.33	52.11 ± 6.60	0.988
IL-23, pg/ml	89.28 ± 5.00	87.96 ± 3.54	0.830	95.05 ± 11.17	85.49 ± 3.89	0.354	77.86 ± 5.22	93.91 ± 4.48	**0.027**

Data presented as mean ± SEM

Abbreviations. *CAS* Cerebral artery stenosis, *AIS* Acute ischemic stroke, *TIA* Transient ischemic attack, *HDL-C* High-density lipoprotein cholesterol, *LDL-C* Low-density lipoprotein cholesterol, *eGFR* Glomerular filtration rate, *HbAc1* Glycosylated hemoglobin A1c

### Higher serum RANTES levels were associated with the presence of CAS

The multivariable logistic regression analysis showed that serum RANTES was independently associated with CAS in all subjects (Table S1). In non-AIS patients, RANTES was significantly associated with CAS (unadjusted OR: 1.169; 95% CI: 1.02**–**1.34; *p* = 0.022 and adjusted OR: 1.041; 95% CI: 1.02**–**1.12; *p* = 0.004). In AIS patients, RANTES was significantly associated with CAS (unadjusted OR: 1.17; 95% CI: 1.03**–**1.33; *p* = 0.017), and the adjusted odds ratio of RANTES was statistically significant (OR: 1.024; 95% CI: 1.008**–**1.041; *p* = 0.003; Table 3).

**Table 3:** Multivariate logistic regression analysis showing the predictors for CAS

	**CAS of non-AIS group**				**CAS of AIS group**		
	**Unadjusted OR**	**Adjusted OR**^**a**^	**Adjusted OR**^**b**^		**Unadjusted OR**	**Adjusted OR**^**a**^	**Adjusted OR**^**b**^
RANTES	1.17 (1.02–1.34) *	1.04 (1.02–1.07) *	1.07 (1.02–1.12) *		1.17 (1.03–1.33) *	1.17 (1.03–1.33) *	1.32 (1.10–1.58) *
IP-10	1.04 (1.00–1.09) *	1.08 (1.02–1.15) *	1.27 (1.08–1.50) *		0.99 (0.96–1.02)	–	–
IL-23	0.99 (0.98–1.01)	–	–		1.03 (1.00–1.05) *	–	–

A backward stepwise selection method in a Multivariate Logistics regression model was performed

OR^a^: adjusted for variables (*P* < 0.05)

OR^b^: adjusted for variables (*P* < 0.05) + age, gender, smoking, drinking, medical histories

Abbreviations: *CAS*: Cerebral artery stenosis,*OR* Odd ratio

^*^p < 0.05

Seventy-eight (61.4%) participants had CAS, including 50 (64.1%) patients with single CAS, 23 (29.5%) patients with 2 CAS, and 5 (6.4%) patients with ≥ 2 CAS (Table S2). RANTES concentration was not significantly different among participants with differing stenosed cerebral vessels. The effect of serum RANTES levels on location and severity of responsible CAS was also analyzed, although was not significantly associated.

## Discussion

In the current study, we demonstrate that patients with AIS have elevated serum RANTES levels compared to patients with non-AIS. Although the AIS and no-AIS subjects were not matched for age, importantly, the multivariate regression analysis excluded the impact of this difference on our results. Interestingly, RANTES appears independently associated with CAS and aids in CAS identification regardless of stroke status. Indeed, the concentration of RANTES as a marker in the atherosclerosis process may distinguish individuals with an increased risk of CAS and may be a potential target for future drug intervention.

Regarding the correlation between RANTES and ischemic stroke, our results are similar to previous studies that the serum RANTES levels increase in AIS [19, 20]. However, the role of RANTES in ischemic stroke remains controversial. As reported, the neurons could produce RANTES which has the potential to directly or indirectly protect neurons by producing neurotrophic factors in the area around the infarct after ischemic stroke, [21] although other studies demonstrate opposite results [22–24]. As a pro-inflammatory chemokine, RANTES could recruit white blood cells to the infarct area and exacerbate cerebral infarction volume. These altering functions may be due to the functional differences of RANTES secreted by different cell types or the varying receptors involved in signal modulation [25]. Further research to clarify its mechanism is warranted.

Atherosclerosis is increasingly recognized as a chronic inflammatory disease. Indeed, inflammation plays an important role in all stages of atherosclerosis and provides a significant pathophysiological mechanism for the formation and rupture of atherosclerotic plaque. Previous studies have reported RANTES in atherosclerotic plaques [26–28] and different cell types, including macrophages, CD8 + T cells, smooth muscle cells and platelets [29, 30]. The expression of CCL5 could promote the proliferation of SMCs and convert them into a synthetic phenotype that causes atherosclerosis, thereby promoting atherosclerosis. Rodent studies have demonstrated RANTES overexpression in atherosclerotic lesions, and genetic deletion of the gene responsible for RANTES receptor CCR5 reduces the atherosclerosis progression [31]. Furthermore, elevated levels of serum RANTES are associated with coronary artery stenosis [31], carotid artery disease and peripheral artery disease. An ARIC carotid MRI study also demonstrated that higher RANTES levels are associated with higher lipid core volume, which may lead to the formation of high-risk plaques [32]. Indeed, higher levels of RANTES were found in patients with symptomatic intracranial arteriosclerosis compared with asymptomatic intracranial arteriosclerosis [33, 34]. Here, we report peripheral serum RANTES levels are closely associated to the presence of CAS, and serum RANTES level effectively identify the presence of CAS. While there was not a significant relationship between CAS characteristics, this may be due to the small sample size, and additional therefore research is warranted.

In this study, we found that in addition to RANTES, other cytokines, such as MMP-1, IP-10 and IL-23, were also different between groups. As previously reported, elevated MMP-1 is associated with AIS [23], while IP-10 and IL-23 are associated with atherosclerosis [35]. This result suggests an inflammatory reaction and accompanied by changes in the expression level of some cytokines regardless of AIS or CAS formation. However, further studies are needed to confirm the stability of this result.

The present study has some limitations. First, this study has a small sample size and is conducted exclusively Chinese patients, which may lead to some selection bias and lack of generalizability outside the Asian population. Therefore, the results need to be interpreted carefully and verified further. Second, the use of antiplatelet drugs and statins after ischemic stroke may affect the concentration of RANTES. However, we minimized the potential effects of stroke and related drugs on RANTES levels by dividing the participants into AIS and non-AIS groups. Finally, we could not present the dynamic change of RANTES levels among patients with acute ischemic stroke in this study since we only measured the serum RANTES levels at a single time point.

## Conclusion

In conclusion, patients with CAS had higher levels of serum RANTES compared to patients with non-CAS, which suggests that the RANTES level might play a role in the physiopathological mechanism of CAS formation, although additional studies are needed to verify this result.

## Authors’contributions

YPG, QQK and YZ. collected the clinical data. YPG, JZ and HH. processed statistical data. YPG, ZYY and DH. drafted and revised the manuscript. XL. designed and guided the study. All authors read and approved the final manuscript.

## Supplementary Information

**Additional file 1:** **Table S1.** Multivariate logistic regression analysis showing the predictors for the CAS. **Table S2.** CAS characteristics of patients with and without AIS

## Data Availability

The data used to support the findings of this study are available from the corresponding author upon request.
