# Supplementary material for: Elevated RANTES levels are associated with increased risk of cerebral atherosclerotic stenosis
Source: BMC Neurol. 2023 Jan 25;23:39. doi: 10.1186/s12883-023-03079-9 (PMC9875531; doi:10.1186/s12883-023-03079-9)
Supplement: Supplementary file 1 — Additional file 1: Table S1. Multivariate logistic regression analysis showing the predictors for the CAS. Table S2. CAS characteristics of patients with and without AIS [file 12883_2023_3079_MOESM1_ESM.docx]

# Supplemental Data

**Table S1. Multivariate logistic regression analysis showing the predictors for the CAS.**

|  | Univariate logistic regression | | | Multivariate logistic regression | | |
| --- | --- | --- | --- | --- | --- | --- |
|  | Unadjusted OR | 95% CI | p-value | Adjusted OR | 95% CI | p-value |
| **CAS of all patients** |  |  |  |  |  |  |
| Age | 0.999 | 0.968–1.031 | 0.958 | – | – | – |
| Male | 1.879 | 0.835–4.230 | 0.128 | – | – | – |
| Smoking | 2.267 | 1.068–4.810 | 0.033 | 2.931 | 1.285–6.685 | 0.011 |
| RANTES | 1.167 | 1.065–1.278 | 0.001 | 1.194 | 1.083–1.317 | 0.000 |

Adjusted OR, adjusted by age, male and variables with a p-value <0.05 by univariate logistic regression. Abbreviations: CAS: cerebral artery stenosis.

**Table S2. CAS characteristics of patients with and without AIS.**

| **CAS Characteristics** | **All patients with CAS (n=78)** | **CAS in non**-**AIS group (n=39)** | **CAS in AIS group (n=39)** |
| --- | --- | --- | --- |
| **Number of CAS** |  |  |  |
| n=1 | 50 (64.1) | 25 (64.1) | 25 (64.1) |
| n=2 | 23 (29.5) | 12 (30.8) | 21 (28.2) |
| n≥3 | 5 (6.4) | 2 (5.1) | 3 (7.7) |
| **Location of responsible CAS** |  |  |  |
| Intracranial only | 41 (52.6) | 19 (48.7) | 22 (56.4) |
| Extracranial only | 31 (39.7) | 16 (41.0) | 15 (38.5) |
| Intracranial and Extracranial | 6 (7.7) | 4 (10.3) | 2 (5.1) |
| **Severity of responsible CAS** |  |  |  |
| 50%–70% | 1 (1.3) | 1(2.6) | 0 (0.0) |
| 70%–99% | 33 (42.3) | 14 (35.9) | 19 (48.7) |
| Occlusion | 44 (56.4) | 24 (61.5) | 20 (51.3) |

Abbreviations: CAS: cerebral artery stenosis; AIS, acute ischemic stroke
